# Intermittent hypoxia differentially affects metabolic and oxidative stress responses in two species of cyprinid fish

**DOI:** 10.1242/bio.060069

**Published:** 2023-09-18

**Authors:** Halina Falfushynska, Inna M. Sokolova

**Affiliations:** ^1^ Department of Marine Biology, Institute for Biological Sciences, University of Rostock, Rostock 18059, Germany; ^2^ Department of Electrical, Mechanical and Industrial Engineering, Anhalt University for Applied Sciences, Köthen 06366, Germany; ^3^ Department of Maritime Systems, Interdisciplinary Faculty, University of Rostock, Rostock 18059, Germany

**Keywords:** Hypoxia, Reoxygenation, Silver carp, Gibel carp, Oxidative stress, Metabolic disorders, Apoptosis, Autophagy

## Abstract

Oxygen fluctuations are common in freshwater habitats and aquaculture and can impact ecologically and economically important species of fish like cyprinids. To gain insight into the physiological responses to oxygen fluctuations in two common cyprinid species, we evaluated the impact of short-term intermittent hypoxia on oxidative stress and metabolic parameters (including levels of prooxidants and oxidative lesions, antioxidants, mitochondrial enzyme activities, mitochondrial swelling, markers of apoptosis, autophagy and cytotoxicity) in silver carp *Hypophthalmichthys molitrix* and gibel carp *Carassius gibelio.* During hypoxia, gibel carp showed higher baseline levels of antioxidants and less pronounced changes in oxidative and metabolic biomarkers in the tissues than silver carp. Reoxygenation led to a strong shift in metabolic and redox-related parameters and tissue damage, indicating high cost of post-hypoxic recovery in both species. Species-specific differences were more strongly associated with oxidative stress status, whereas metabolic indices and nitrosative stress parameters were more relevant to the response to hypoxia-reoxygenation. Overall, regulation of energy metabolism appears more critical than the regulation of antioxidants in the response to oxygen deprivation in the studied species. Further research is needed to establish whether prioritizing metabolic over redox regulation during hypoxia-reoxygenation stress is common in freshwater cyprinids.

## Teaser

**Summary:** This research sheds light on oxidative stress and metabolic adjustments to oxygen fluctuations on two common species of freshwater cyprinids.

## INTRODUCTION

Dissolved oxygen (DO) concentrations are a major factor influencing biological diversity and productivity of freshwater ecosystems worldwide. Since the industrial revolution, hypoxia (oxygen deficiency) has been spreading in freshwater habitats threatening biodiversity, ecosystem functions and services (Jenny et al., 2016). Eutrophication due to the nutrient pollution is the main driver of aquatic hypoxia (Smith and Schindler, 2009), and its effects are amplified by global climate change that leads to warming of the surface waters, stimulates microbial respiration, decreases oxygen solubility and enhances stratification in the lakes and coastal areas (Jenny et al., 2016). Freshwater fish are sensitive organisms to hypoxia owing to their relatively high oxygen demand and the physiological limitations of oxygen uptake in water (Rubalcaba et al., 2020). Oxygen deficiency is one of the major drivers of massive fish kills (La and Cooke, 2011), and even milder sublethal hypoxia can negatively impact fish growth, behavior, physiology and immunity, leading to cascading effects in freshwater ecosystems (Hrycik et al., 2017; Pollock et al., 2007). Hypoxia is also a major concern for aquaculture negatively impacting the production and sustainability of land-locked and coastal aquaculture (Dong et al., 2011).

Oxygen fluctuations have multiple negative effects on the metabolic and redox homeostasis of animals including fish. Oxygen deficiency can result in the low ATP output due to a decrease in aerobic ATP generation and increased reliance on the less efficient anaerobic glycolysis (Kalogeris et al., 2014). Suppressed aerobic metabolism can lead to a mismatch between the cellular ATP demand and supply and impair vital ATP-demanding functions such as ion or protein homeostasis (Wheaton and Chandel, 2011). Furthermore, hypoxia and especially subsequent reoxygenation represent major oxidative stress caused by the electron leak from reduced redox carriers in the cell and elevated production of reactive oxygen and nitrogen species (ROS and RNS) (Boutilier, 2001; Sgarbi et al., 2018). An excess of ROS upregulates antioxidant signaling pathways leading to compensatory increase in antioxidant enzymes and ROS scavengers (such as glutathione) (Schieber and Chandel, 2014). Thus, in a largemouth bass *Micropterus salmoides* short-term hypoxic stress (<40 min at 3-4 mg O_2_ L^−1^) led to a significant upregulation of oxygen- and redox-sensitive transcription factors (Hypoxia-inducible factor 1-alpha HIF-1α, Nuclear factor erythroid 2-related factor 2 Nrf2, and kelch-1ike ECH- associated protein l Keap1) and elevated expression of catalase and glutathione peroxidase*,* but suppressed the expression of superoxide dismutase (Xin et al., 2022). The upregulation of antioxidants is considered important for prevention of the oxidative damage to proteins, lipids, and DNA during hypoxia (Hermes-Lima et al., 2015; Xin et al., 2022) but can incur significant energy cost to the organism (Hou and Amunugama, 2015; McWilliams et al., 2020). A need for the energy investment into the cellular antioxidant protection might conflict with the necessity to conserve energy during hypoxia in fish (Buck and Hochachka, 1993; Hochachka, 1995; Hou and Amunugama, 2015). Therefore, integrated assessments of bioenergetics and oxidative stress responses during hypoxia and recovery are required to advance our understanding of the cellular mechanisms that contribute to hypoxia sensitivity and tolerance in fish.

Cyprinids are ecologically important in freshwater ecosystems, extensively farmed around the world and include some important biomedical model species such as zebrafish (Orban and Wu, 2008; Winfield and Nelson, 1991). Species of cyprinids widely vary in their tolerance to abiotic stressors including hypoxia (Winfield and Nelson, 1991). Some species such as the goldfish (*Carassius auratus*) and crucian carp (*Carassius carassius*) are among the vertebrate champions of hypoxia tolerance and can withstand extended periods of anoxia, whereas others such Chinese hook snout carp (*Zacco platypus*) or sharp-jaw barbell (*Onychostoma sima*) are sensitive to oxygen deficiency (Bickler and Leslie, 2007; Fu et al., 2014). Variation in hypoxia tolerance among cyprinids has been linked with the ability to prevent lactate accumulation by converting lactate into easily excretable ethanol, the amount of glycogen stores that serve as metabolic fuel during anoxia and the ability to remodel gills in response to hypoxia (Dhillon et al., 2013; Fu et al., 2014; He et al., 2015). However, the role of susceptibility to oxidative stress in response to oxygen fluctuations has not been extensively studied in cyprinids except in the extremely tolerant model species such as the crucian carp and the goldfish (Bagnyukova et al., 2005; Lushchak and Bagnyukova, 2006b,a; Lushchak et al., 2001). Studies in hypoxia-intolerant terrestrial vertebrates suggest that control over oxidative damage plays a key role in preventing cellular and tissue injury during oxygen fluctuations (de Vries et al., 2013; Wu et al., 2020). If similar mechanisms play a role in hypoxia-tolerant cyprinids, we anticipate higher upregulation of antioxidants, lower levels of oxidative injury and less cellular damage (e.g. lower levels of autophagy and apoptosis) during hypoxia and recovery. Alternatively, if energy conservation has priority for hypoxia response, fish might show an improved metabolic response and little change in the redox status during hypoxia and reoxygenation.

To test these hypotheses, we exposed two species of cyprinids, gibel carp *Carassius gibelio* (Bloch, 1782) and silver carp *Hypophthalmichthys molitrix* (Valenciennes, 1844) to short-term (1 h) hypoxia (2.0 mg O_2_ L^−1^) followed by 1 h of normoxic recovery, and determined the shifts in the antioxidant parameters (including the total antioxidant capacity, activities of catalase, glutathione-S-transferase and glutathione levels) and oxidative stress markers (levels of reactive oxygen and nitrogen species, lipid peroxidation, protein carbonylation and DNA strand breaks) in the gills and the liver of the hypoxia-reoxygenation (H/R) exposed fish. To assess the impacts of H/R stress on aerobic metabolism, we measured activities of key mitochondrial enzymes (succinate dehydrogenase and cytochrome c oxidase) and mitochondrial swelling in the fish tissues. To assess the potential cytotoxic effects of H/R stress, we measured the activity of a key executor caspase (caspase 3) that plays an important role in apoptosis, in the liver and the gill and cathepsin D activity as a marker of autophagy in the liver. As general cyto- and neurotoxicity markers, lysosomal membrane stability was measured in the liver, the lactate dehydrogenase activity was assessed in the blood plasma and the activity of acetylcholine esterase was measured in the brain. The two cyprinid species chosen as models for this research are broadly distributed and commonly farmed freshwater species in Europe and the world. Gibel carp *C. gibelio* is often stocked in aquaculture together with the common carp *Cyprinus carpio* and has widely spread throughout European freshwaters. *C. gibelio* is among the most hypoxia-tolerant cyprinid species adapted to slow flowing and stagnant waters (Kottelat and Freyhof, 2007). Silver carp *H. molitrix*, a native of east Asia, has been introduced around the world for aquaculture and control of algal blooms (Li and Fang, 1990). This fish typically inhabits moderate-flow rivers, possesses gill remodeling ability similar to *Carassius gibelio*, but shows higher sensitivity to severe hypoxia (∼0.3 mg O_2_ L^−1^ for 48 h) based on the response of loss of equilibrium and aquatic surface respiration (Fu et al., 2014; Radke and Kahl, 2002). The comparative analysis of the metabolic, oxidative and nitrosative stress parameters and potential cytotoxic outcomes during H/R stress thus provides insights into the molecular and cellular mechanisms that might contribute to differences in responses to hypoxia-reoxygenation and have implications for understanding the potential impacts of oxygen fluctuations in the fish farms and native habitats on these two widespread fish species.

## RESULTS

Most of the metabolic, oxidative stress and cytotoxicity parameters we studied showed significant interactive effects of the species and H/R exposures (Table S1), indicating that the responses of these variables to the H/R stress differed between the two species studied. The exceptions were TAC in the liver, the frequency of DNA strand breaks and protein carbonyl levels in the liver and the gills, and acetylcholinesterase activity in the brain that were significantly affected by the biological species, and ROS and total GSH levels in the gills that showed effects of the species and H/R stress, but not their interactions, and mitochondrial swelling in the liver that showed only the effect of H/R stress (Table S1). Total GSH and SDH activity in the liver did not show significant effects of the species or H/R exposures (Table S1).

### Antioxidants

**Fig. 1.**
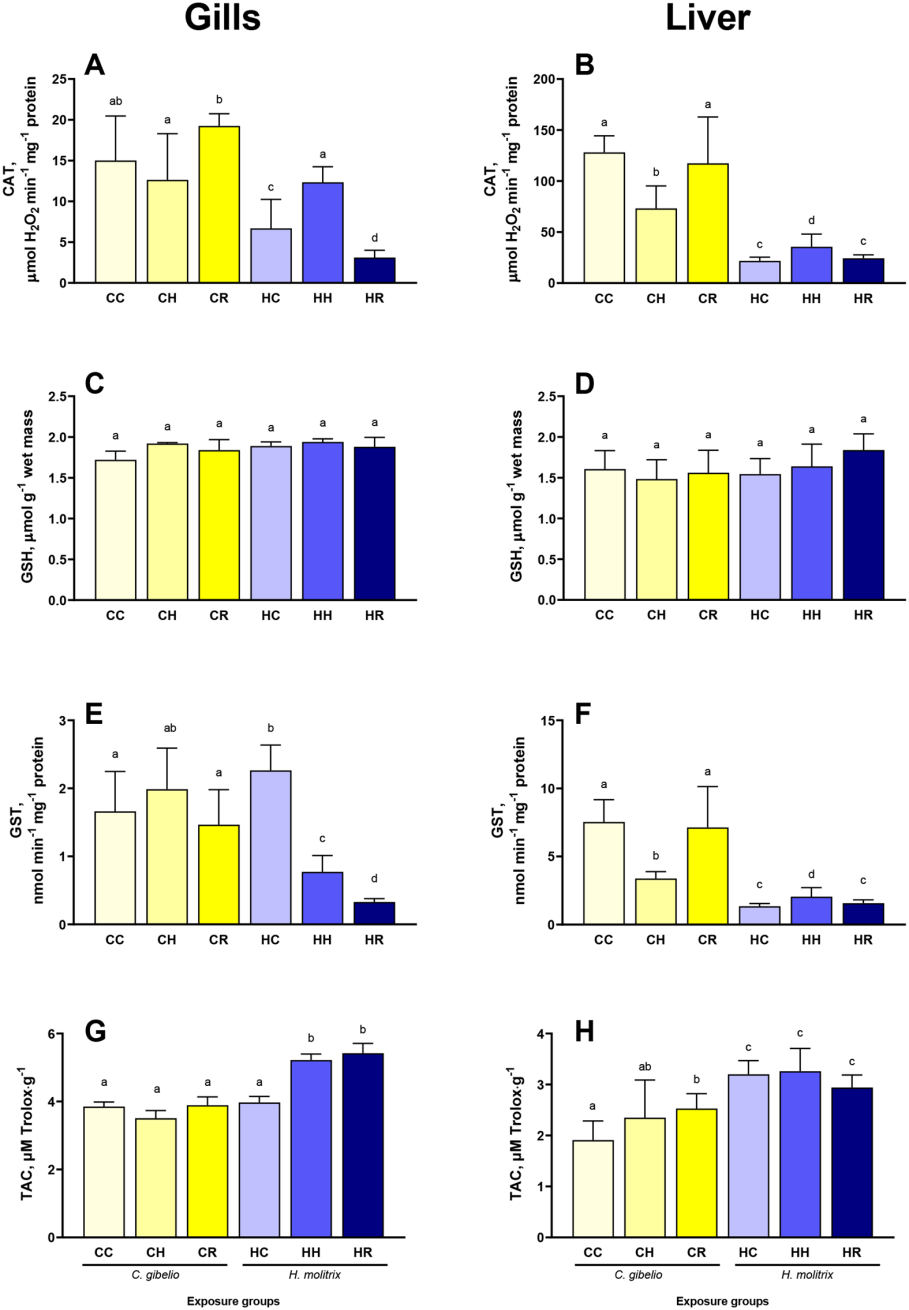
**Effects of hypoxia and reoxygenation stress on antioxidants in the gills and liver of gibel carp *Carassius gibelio* and silver carp *Hypophthalmichthys molitrix***. (A,B) catalase activity; (C,D) total glutathione concentration; (E,F) glutathione-S-transferase activity; (G,H) total antioxidant capacity. Fish were exposed to normoxia (DO=7.5 mg L^−1^, CC and CH groups), hypoxia (DO=2.0 mg L^−1^, CH and HH groups), and reoxygenation (CR and HR groups). Columns that do not share the letters represent significantly different values (*P*<0.05). The means and the standard errors of the mean are shown. *N*=5.

The baseline activity of catalase (CAT) was higher in the gills and the liver of gibel carp compared to silver carp (Fig. 1A,B). In gibel carp, hypoxia exposure had no effect on the CAT activity in the gills but led to a decrease of the CAT activity in the liver (Fig. 1A,B). During reoxygenation, the baseline levels of CAT was restored in the liver of gibel carp (Fig. 1B). In silver carp, CAT activity was upregulated by the hypoxia exposure in the gills and the liver. During reoxygenation, CAT activity decreased below the baseline in the gill and returned to the baseline levels in the liver of silver carp (Fig. 1A,B).

Total glutathione concentrations were similar in the gills and the liver of the two fish species and did not change in response to hypoxia and reoxygenation (Fig. 1C,D).

Gibel carp showed lower baseline glutathione-S-transferase (GST) activity in the gill and higher GST activity in the liver compared with the respective tissue-specific values of silver carp (Fig. 1E,F). The GST activity remained stable during hypoxia in the gills but decreased in the liver of gibel carp. Reoxygenation restored the baseline activity of GST in gibel carp tissues (Fig. 1E,F). In silver carp, the GST activity was strongly suppressed by the hypoxia in the gills and slightly but significantly upregulated in the liver (Fig. 1E,F). During reoxygenation, the GST activity in the gills of silver carp was further suppressed but it returned to the baseline levels in the liver (Fig. 1E,F).

The baseline total antioxidant capacity (TAC) was similar in the gills of the two species but higher in the liver of silver carp relative to that of gibel carp (Fig. 1G,H). The tissue-specific responses of the TAC to H/R stress differed between the two species. Thus, the TAC of the gill tissues remained stable during the hypoxia-reoxygenation exposure in gibel carp but was upregulated in silver carp (Fig. 1G). The TAC of the liver tissue increased during hypoxia and recovery in gibel carp and remained unchanged in silver carp (Fig. 1H).

### Reactive oxygen and nitrogen species

**Fig. 2.**
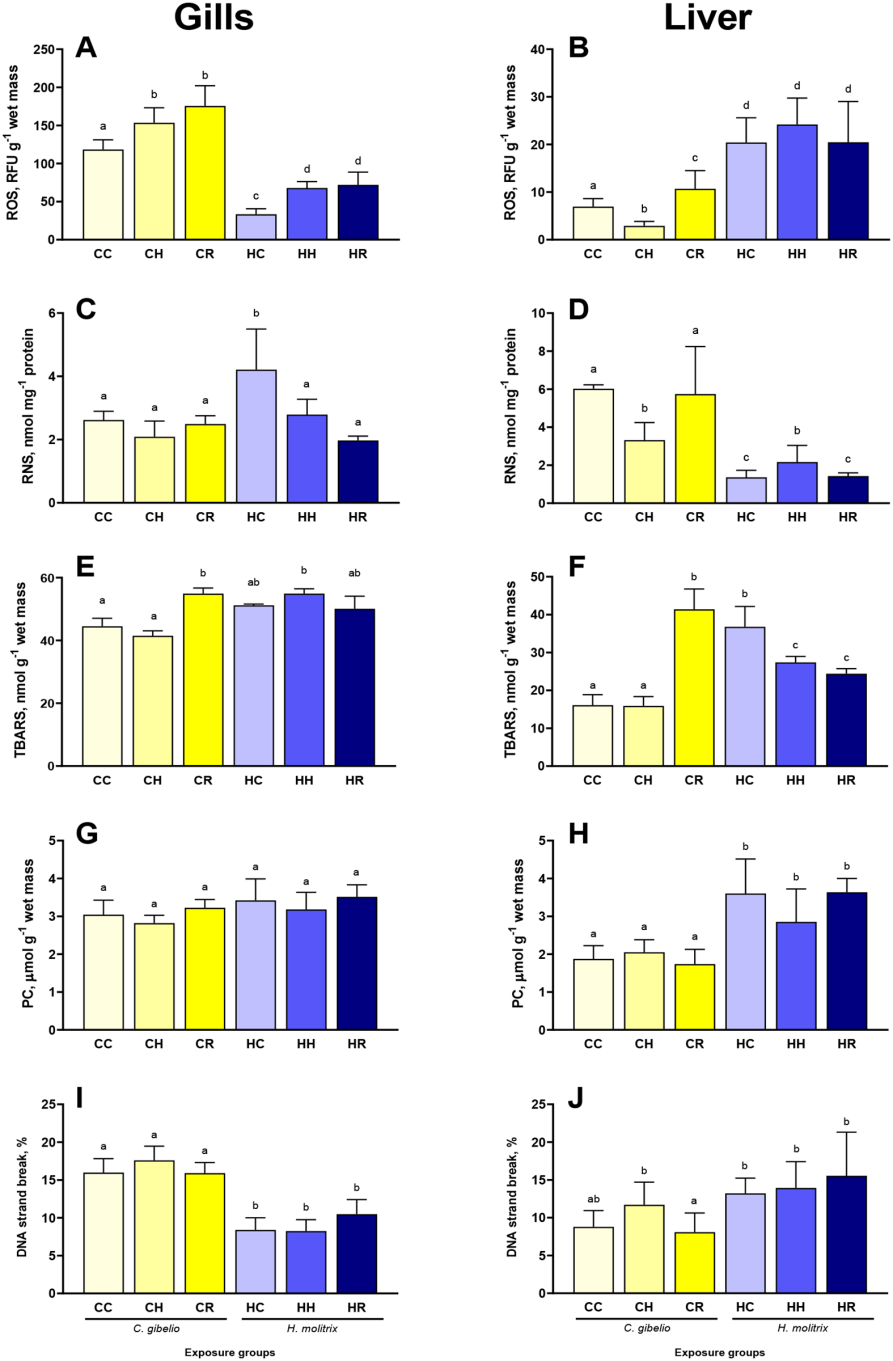
**Effects of hypoxia and reoxygenation stress on radicals and oxidative damage products in the gills and liver of gibel carp *Carassius gibelio* and silver carp *Hypophthalmichthys molitrix*.** (A,B) reactive oxygen species; (C,D) reactive nitrogen species; (E,F) Thiobarbituric acid-reactive substances; (G,H) protein carbonyls; (I,J) DNA – strand breaks. Fish were exposed to normoxia (DO=7.5 mg L^−1^, CC and CH groups), hypoxia (DO=2.0 mg L^−1^, CH and HH groups), and reoxygenation (CR and HR groups). Columns that do not share the letters represent significantly different values (*P*<0.05). The means and the standard errors of the mean are shown. *N*=5.

The baseline levels of ROS generation in the gill tissues were higher in gibel carp than in silver carp (Fig. 2A). Hypoxia and reoxygenation led to a significant increase of the ROS levels in the gills of both studied species although the relative increase was higher in the gills of silver carp (∼twofold) than in gibel carp (∼30-50% increase) (Fig. 2A).

The baseline ROS levels in the liver of gibel carp were lower than in silver carp liver (Fig. 2B). Hypoxia suppressed the ROS levels in gibel carp liver by ∼2.4-fold (Fig. 2B). During reoxygenation, ROS levels increased by ∼50% above the normoxic baseline in gibel carp liver. In silver carp, the ROS levels in the liver did not change in response to hypoxia and reoxygenation (Fig. 2B).

The baseline levels of RNS in the gill tissues were lower in gibel carp then in silver carp; the opposite pattern was seen in the liver tissue (Fig. 2C,D). In gibel carp, the levels of RNS in the gills were not affected by the H/R stress (Fig. 2C). In the gills of silver carp, the RNS levels decreased during hypoxia and reoxygenation by ∼1.5 and 2.1-fold, respectively (Fig. 2C).

In the liver of gibel carp, the RNS levels decreased by ∼1.8-fold during hypoxia and returned to the baseline levels during reoxygenation (Fig. 2D). In silver carp liver, hypoxia led to a modest increase in the RNS levels (by ∼1.6-fold) that returned to the baseline during reoxygenation (Fig. 2D).

### Oxidative lesions

The tissue levels of TBARS (indicative of the lipid peroxidation, LPO) were not affected by hypoxia in the gills or the liver of gibel carp, but increased in both studied tissues during reoxygenation (Fig. 2E). In silver carp, the TBARS levels in the gills were not affected by the H/R stress whereas in the liver the TBARS levels decreased during exposure to hypoxia and subsequent reoxygenation (Fig. 2F).

The tissue levels of the protein carbonyls or the % of DNA strand break did not change in response to the H/R stress in the gills or the liver of the two fish species (Fig. 2G-J).

### Mitochondrial markers

**Fig. 3.**
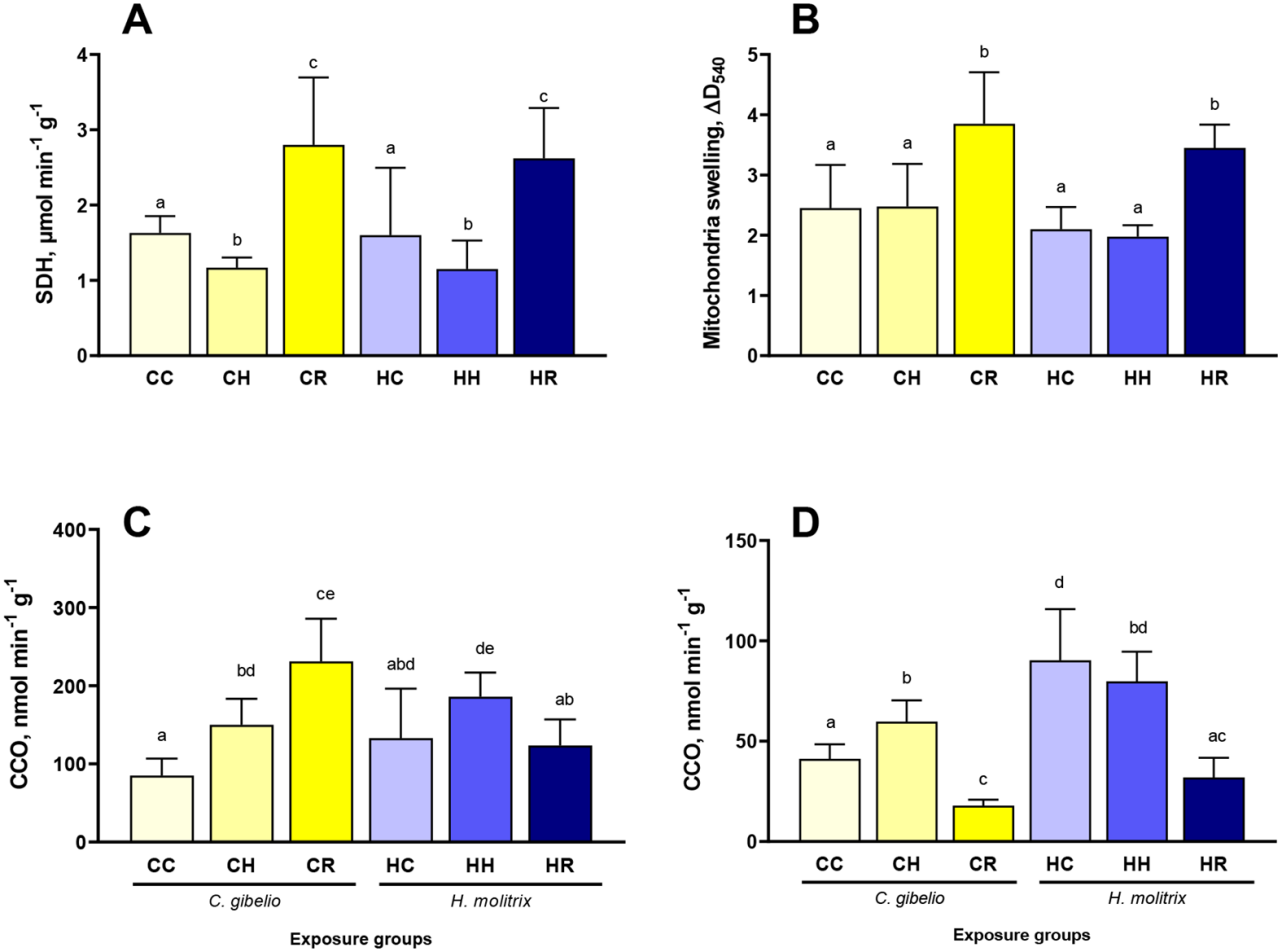
**Effects of hypoxia and reoxygenation stress on mitochondrial traits in the liver (A,B,D) and gills (C) of gibel carp *Carassius gibelio* and silver carp *Hypophthalmichthys molitrix*.** (A) Succinate dehydrogenase activity, (B) mitochondria swelling; (C,D) cytochrome c oxidase activity. Fish were exposed to normoxia (DO=7.5 mg L^−1^, CC and CH groups), hypoxia (DO=2.0 mg L^−1^, CH and HH groups), and reoxygenation (CR and HR groups). Columns that do not share the letters represent significantly different values (*P*<0.05). The means and the standard errors of the mean are shown. *N*=5.

Post-hypoxic reoxygenation (but not hypoxia) led to significant mitochondrial swelling in the liver tissues of both studied species (Fig. 3A).

In the liver of gibel and silver carp, exposure to hypoxia led to a significant decrease in the activity of SDH (mitochondrial Complex II) with subsequent elevation during reoxygenation (Fig. 3B). The SDH activity could not be measured in the gills due to the lack of sample.

Activity of CCO (mitochondrial Complex IV) in the gills of gibel carp was elevated during exposure to hypoxia and further increased upon reoxygenation (Fig. 3C). In the liver of gibel carp, CCO activity also increased during hypoxia but decreased during reoxygenation (Fig. 3D). In silver carp, CCO activity in the gills didn't vary significantly during H/R stress (Fig. 3C). In the liver of silver carp, the CCO activity did not change during hypoxia but was suppressed upon reoxygenation (Fig. 3D).

### Apoptotic and autophagy markers

**Fig. 4.**
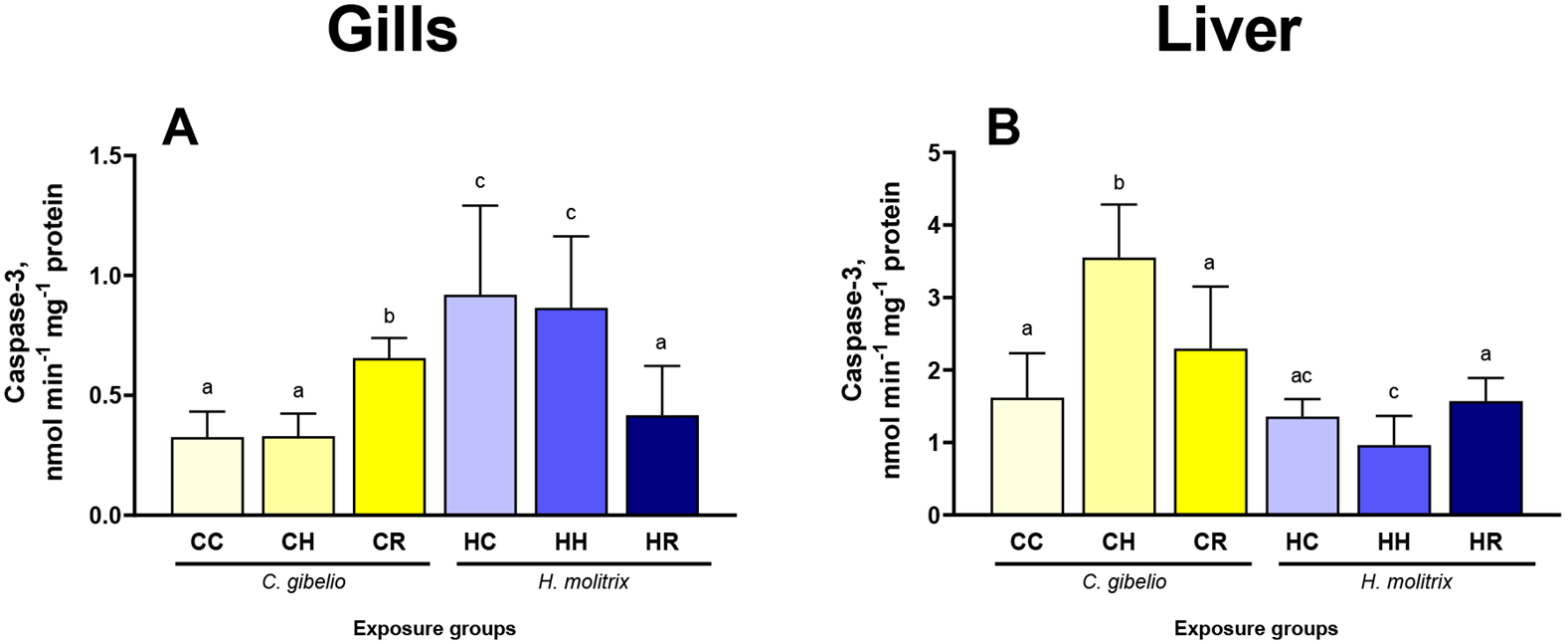
**Effects of hypoxia and reoxygenation stress on caspase-3 activity in the gills and liver of gibel carp *Carassius gibelio* and silver carp *Hypophthalmichthys molitrix*.** Fish were exposed to normoxia (DO=7.5 mg L^−1^, CC and CH groups), hypoxia (DO=2.0 mg L^−1^, CH and HH groups), and reoxygenation (CR and HR groups). Columns that do not share the letters represent significantly different values (*P*<0.05). The means and the standard errors of the mean are shown. *N*=5.

Caspase 3 activity in the gills of the two studied species remained unchanged during hypoxia (Fig. 4A). During reoxygenation, the caspase 3 activity in the gill was elevated in gibel carp and suppressed in silver carp (Fig. 4A). In the liver of gibel carp, the caspase 3 activity was elevated during hypoxia and returned to the baseline during reoxygenation (Fig. 4B). In silver carp, the caspase 3 activity in the liver did not change during hypoxia and reoxygenation (Fig. 4B).

**Fig. 5.**
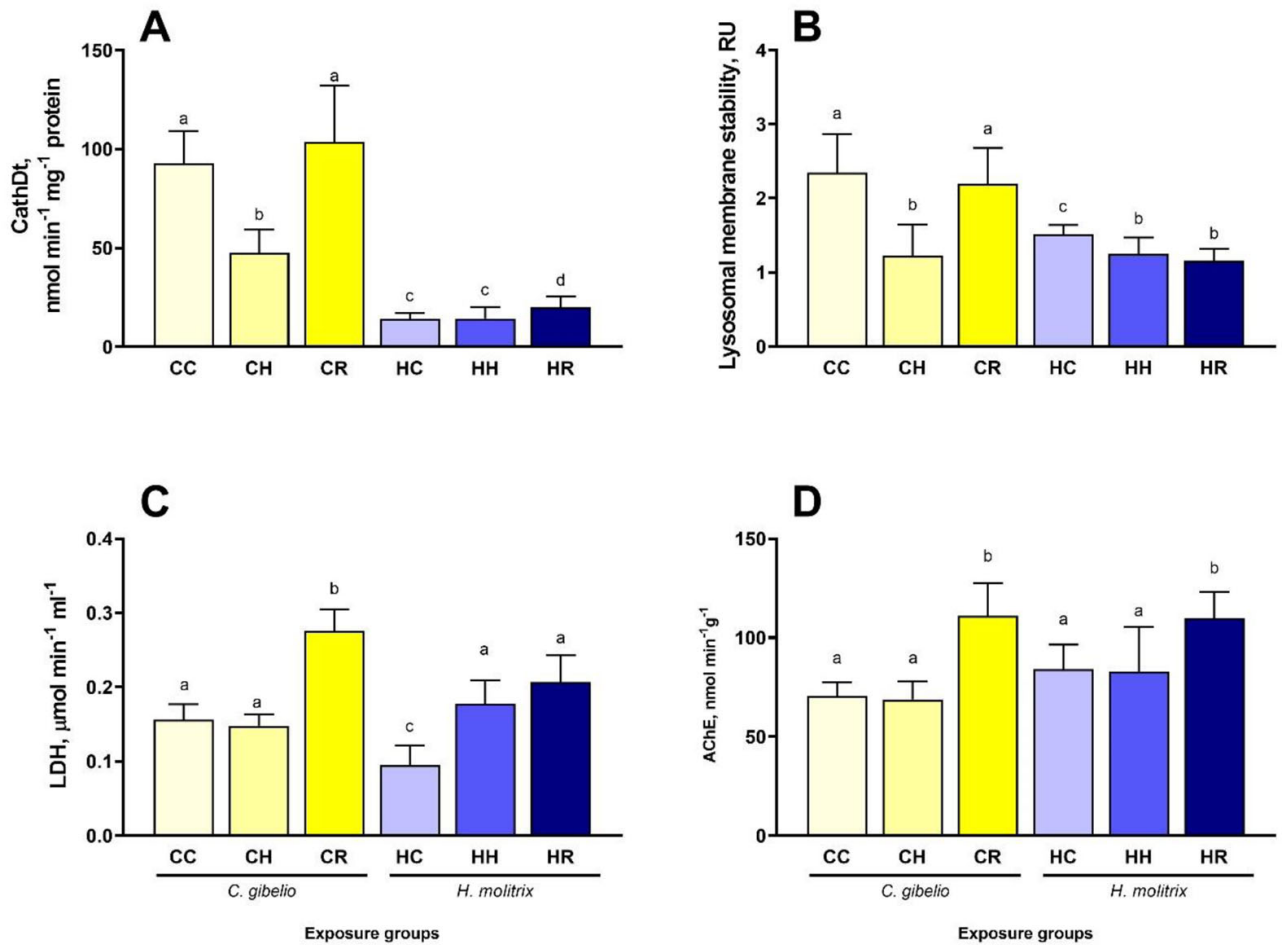
**Effects of hypoxia and reoxygenation stress on autophagy and cytotoxicity markers in the liver (A,B), blood plasma (C), and brain (D) of gibel carp *Carassius gibelio* and silver carp *Hypophthalmichthys molitrix*.** (A) cathepsin D activity, (B) lysosomal membrane stability, (C) lactate dehydrogenase activity, (D) acetylcholine esterase activity. Fish were exposed to normoxia (DO=7.5 mg L^−1^, CC and CH groups), hypoxia (DO=2.0 mg L^−1^, CH and HH groups), and reoxygenation (CR and HR groups). Columns that do not share the letters represent significantly different values (*P*<0.05). The means and the standard errors of the mean are shown. *N*=5.

The baseline activity of cathepsin D activity, the most abundant lysosomal endopeptidase, was considerably higher in the liver of gibel carp than in the liver of silver carp (Fig. 5A). In gibel carp, cathepsin D activities in the liver significantly decreased during hypoxia and returned to the baseline during reoxygenation (Fig. 5A). In silver carp liver, cathepsin D activities did not change in hypoxia but strongly increased during reoxygenation (Fig. 5A).

Lysosomal membrane stability decreased during hypoxia in the liver of both studied fish species and recovered during reoxygenation in gibel but not in silver carp (Fig. 5B).

### Tissue-damage markers

In gibel carp, the blood plasma LDH levels (indicative of the cell damage) remained stable during hypoxia exposure and increased significantly during reoxygenation (Fig. 5E). In silver carp, the plasma LDH activity increased during hypoxia and remained elevated during reoxygenation (Fig. 5C).

Acetylcholinesterase activity in the brain was not affected by hypoxia but increased during reoxygenation in both studied fish species (Fig. 5D).

### Multivariate analyses

**Fig. 6.**
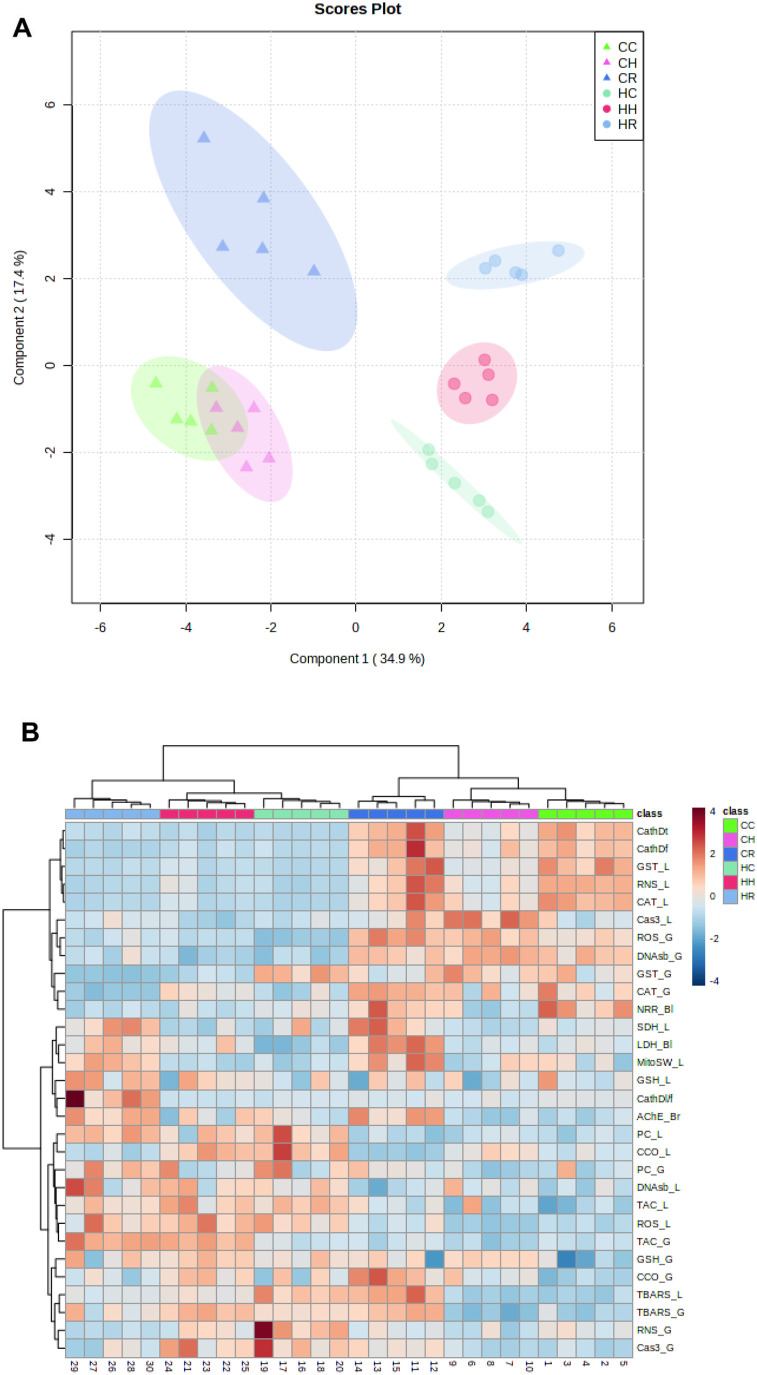
Score biplot of partial least square discriminant analysis (A) and heat map analysis (B) of the studied biological traits of gibel carp and silver carp exposed to normoxia, hypoxia, and reoxygenation.

PLS-DA analyses based on all studied physiological and biochemical trait showed a good separation of the biochemical profiles of the two species studied along the first component (34.9% of data variation), and the treatment conditions within the same species – along the second component (17.4% of the data variation) (Fig. 6A). The first (species-specific) PLS-DA axis had high loadings (the absolute values >0.2) of the TAC, catalase activity and ROS levels in both studied tissues, total and free cathepsin D activity, RNS and GST activity in the liver, and protein carbonyl levels and DNA strand breaks in the gill (Table S2). The second (oxygen regime) axis had high loadings of the mitochondrial SDH, CCO activity and swelling in the liver, LDH levels in the blood, AChE activity in the brain, and RNS levels and GST activity in the gills (Table S1). Notably, the biochemical tissue profile of the more hypoxia tolerant gibel carp did not change during hypoxia exposure but strongly shifted during the normoxic recovery (Fig. 6A). In silver carp, the biochemical tissue profile shifted in response to hypoxia and further shifted along the same axis during reoxygenation (Fig. 6A).

The heat-map analysis showed strong clustering of the samples according to the species as well as the oxygen regimes indicating different patterns of responses to hypoxia-reoxygenation between the studied species (Fig. 6B). One cluster involved the variables that showed low baseline levels and little change in response to hypoxia-reoxygenation in silver carp, but higher baseline and variability in response to the oxygen regime in gibel carp. This cluster included cathepsin D, catalase, GST and caspase 3 activities, RNS levels, and NRR in the liver; catalase and GST activities, ROS and DNA strand break levels in the gills (Fig. 6B). These parameters were generally lower in silver carp compared to gibel carp and did not change in response to hypoxia and reoxygenation (Fig. 6B). The second large cluster involved traits that were initially higher and/or increased during hypoxia in silver but not in gibel carp, including protein carbonyl and LPO levels, TAC and CCO activity, in the liver and the gill, RNS, GSH and caspase 3 activity in the gill and ROS levels in the liver (Fig. 6B). The remaining cluster involved traits that had relatively high baseline levels in both studied species and decreased during the H/R stress including SDH activity and mitochondrial swelling in the liver, GSH and cathepsin D activity in the liver, blood LDH levels and AChE activity in the brain (Fig. 6B).

## DISCUSSION

### Species-specific versus H/R-induced variation in metabolic and redox parameters

In this study, we examined the impact of hypoxia-reoxygenation (H/R) stress on two cyprinid species, gibel carp and silver carp, and found significant species-specific differences in their tissue-level metabolic and redox-related traits. Our results showed that species specificity was the primary determinant (>34% of variation) of the tissue profiles of all studied metabolic and redox parameters (as shown in Fig. 6). Oxidative-stress related markers (TAC and ROS levels in the gill and liver, RNS, CAT and GST in the liver, and DNA strand break in the gill) and the autophagy marker (cathepsin D activity in the liver) were the parameters most strongly associated with PLS-DA Component 1, which separated the two studied cyprinid species. Gibel carp exhibited higher baseline activities of antioxidant enzymes, including catalase (CAT) and glutathione S-transferase (GST), in both gill and liver tissues compared to silver carp. In contrast, the baseline concentrations of reduced glutathione (GSH) and total antioxidant capacity (TAC) were similar in both studied species. Notably, lower baseline activities of enzymatic antioxidants in silver carp corresponded with higher levels of reactive oxygen species (ROS), lipid peroxides, and carbonyls, especially in the liver. These differences in redox-related parameters might be attributed to the species-specific lifestyles and habitats of these two cyprinid species. Gibel carp, as a benthic fish adapted to live in stagnant and polluted waters, may have developed more effective defenses against oxidative stress and improved detoxification mechanisms to prevent the accumulation of oxidative damage compared to the more pelagic silver carp (Fu et al., 2014; Kottelat and Freyhof, 2007; Radke and Kahl, 2002). This is likely due to the higher exposure of gibel carp to environmental stressors, including hypoxia and pollutants. On the other hand, silver carp demonstrated a higher aerobic capacity, as indicated by higher cytochrome c oxidase (CCO) activity in the gill and liver tissues, in line with its more active and aerobic lifestyle (Fu et al., 2014; Kottelat and Freyhof, 2007; Radke and Kahl, 2002).

A comparative analysis of the metabolic and redox-related markers in gibel carp and silver carp revealed complex and species-specific response to hypoxia and reoxygenation. Thus, exposure to hypoxia resulted in a modest alteration in the tissue profiles of these markers in both the gill and liver tissues of gibel carp, as compared to the normoxic control state. However, during reoxygenation, a significant shift in these biomarker profiles was observed, indicating a robust metabolic and redox reorganization during recovery. Conversely, in silver carp, hypoxia exposure led to a marked shift in the tissue profiles of metabolic and oxidative stress-related markers, which was further exacerbated during reoxygenation. These observations suggest a more severe response to hypoxia in silver carp compared to gibel carp. The Component 2 in the PLS-DA analysis (which separated the groups exposed to different oxygen regimes and explained approximately 17% of the overall biomarker variation) was associated primarily with metabolic parameters such as CCO and SDH activity in the liver, mitochondrial swelling response, tissue damage markers (plasma LDH levels and AChE activity in the brain), and RNS and GST levels in the gill. These findings suggest that the regulation of energy metabolism is more critical than the regulation of antioxidants in the response to H/R stress in both gibel carp and silver carp.

### Effects of hypoxia and reoxygenation on the oxidative stress in cyprinids

Our results showed that in gibel carp, exposure to hypoxia did not activate the antioxidant system in the gill or liver. Specifically, the levels of total antioxidant capacity (TAC) and the activities of catalase (CAT) and glutathione S-transferase (GST) remained at baseline levels in the gill, while in the liver, the activities of CAT and GST were suppressed, despite no change in TAC. This suppression of enzymatic antioxidants in the liver may be attributed to the decrease in reactive oxygen species (ROS) and reactive nitrogen species (RNS) levels during hypoxia, which could reflect a metabolic rate suppression - a common adaptive strategy in hypoxia-tolerant species (Bickler and Leslie, 2007). On the other hand, silver carp showed an activation of the antioxidant defense system in response to hypoxia exposure, with an increase in TAC and CAT activity in both the gill and liver, along with higher GST activity in the liver. Furthermore, the increase in antioxidant defense in the gill was associated with elevated ROS levels, while the increase in the liver was not linked to changes in ROS levels. Interestingly, our findings revealed concordant variations in GST activity and RNS levels in response to hypoxic conditions in both species, in line with the proposed function of GST in nitric oxide regulation (Aravilli et al., 2021; Russell and Richardson, 2022). Neither of the two studied cyprinid species exhibited an increase in oxidative damage to their membranes, proteins, or DNA, suggesting that alterations in ROS and RNS levels caused by hypoxia remain manageable by the antioxidant system's capabilities.

During post-hypoxic recovery, gibel carp exhibited a return to baseline CAT and GST activity levels in their tissues despite a significant increase in ROS levels. However, this coincided with an increase in TBARS levels, indicating oxidative damage to cellular membranes. Although the liver showed a slight increase in TAC levels during reoxygenation, it was not enough to prevent TBARS accumulation. In contrast, the hypoxia-induced CAT and GST activities in silver carp decreased during reoxygenation, dropping to or below the normoxic baseline levels in the liver and gill, respectively. Like gibel carp, silver carp experienced decreased enzymatic antioxidant activity during reoxygenation, despite increased ROS levels in the gill. However, no TBARS accumulation was found in silver carp, and oxidative damage to proteins and DNA remained at background levels during reoxygenation in both species. These findings suggest that the variation in redox-related parameters induced by H/R, including antioxidant activities, ROS and RNS levels, and oxidative lesions, does not clearly correlate with the differences in hypoxia tolerance between the two studied species. While the more hypoxia-tolerant gibel carp maintained tissue redox status during hypoxia with stable or decreasing levels of pro- and antioxidants, they were susceptible to oxidative damage to membranes during reoxygenation. In contrast, silver carp showed considerable variability in redox-related parameters during hypoxia and recovery but was still able to prevent oxidative damage to membranes, proteins, and DNA under all experimental conditions. Several other studies have reported only slight differences in redox-related parameters, such as the activities of catalase, superoxide dismutase, glutathione-S-transferase, glutathione peroxidase, tissue levels of ROS, and lipid peroxidation products, in response to hypoxia-reoxygenation stress in fish (Lushchak et al., 2001; Castro et al., 2020; Xin et al., 2022; Leveelahti et al., 2014; Wischhusen et al., 2020). This suggests that the ability to control oxidative stress and prevent accumulation of oxidative damage during oxygen fluctuation is not a reliable predictor of hypoxia tolerance in these species.

### Effects of hypoxia and reoxygenation on cellular damage markers of cyprinids

Membrane stability of cellular organelles is an essential parameter for health of aquatic animals as well as a predictive tool to evaluate the toxic effects of xenobiotics and abiotic stressors including oxygen deprivation (Falfushynska et al., 2020, 2022). Stress-induced lysosome and mitochondrial damage can result in alterations in the energetic budget, cell damage, apoptosis, and tissue inflammation (Weinberg et al., 2000; Niquet et al., 2003; Chiche et al., 2010; Fang et al., 2011; Troncoso et al., 2018; Hong et al., 2019). As an example, early mitochondrial swelling and loss of the mitochondrial membrane potential associated with lower activity of respiratory complexes led to poor cell growth, decreased cellular respiration, and caspase-3-mediated cell death in hypoxic neurons (Niquet et al., 2003; Chen et al., 2005). Being connected to the severity of hypoxia exposure, lysosomal and mitochondrial membrane integrity might serve as a useful indicator of cellular damage caused by hypoxia in fish.

Our studies have shown that gibel carp responded to oxygen deficiency by downregulating autophagic enzyme activity, as evidenced by a decrease in total cathepsin D activity. In addition, there was a decrease in lysosomal integrity and an upregulation of caspase 3, an important apoptotic executioner caspase in the liver. Despite this shift in autophagy- and apoptosis-related activities, gibel carp does not exhibit systemic tissue damage during hypoxia, as indicated by low background LDH activity in the blood plasma. During reoxygenation, all studied cytological damage markers returned to baseline levels in gibel carp liver. However, caspase 3 activity was upregulated in the gill, and plasma LDH levels increased, indicating tissue damage. Since LDH activity in plasma integrates cellular damage signals from multiple organs and tissues it is impossible to say which organ sustained damage in gibel carp during reoxygenation. Nevertheless, these data, similar to the findings on redox-related parameters, suggest that reoxygenation is more physiologically challenging to gibel carp than hypoxia. Unlike gibel carp, silver carp showed signs of tissue damage (indicated by elevated plasma LDH levels) and decrease in the lysosomal membrane integrity in hypoxia with no signs of recovery but slight activation of autophagy (cathepsin D activity) during reoxygenation.

The relationship between autophagy and apoptosis during oxygen deprivation is a topic of great importance, but it is often overlooked, especially in lower vertebrates. Recent studies have shed light on the role of autophagy and apoptosis in response to different levels of stress. While early induction of autophagy may act as a protective response, prolonged autophagy can lead to cell death (Azad et al., 2008). Gibel carp was able to tackle H/R stress by decreasing autophagic enzyme activity but upregulating caspase 3 and apoptotic-related genes including *bax, casp3*, *casp9* and *ero1α* (Wu et al., 2022) during hypoxia, which may serve as a protective mechanism against tissue damage. Silver carp, on the other hand, showed only a slight activation of autophagy during reoxygenation and no sign of recovery, suggesting that hypoxia is a more significant physiological challenge for this species than it is for gibel carp.

Interestingly, brain levels of acetylcholine esterase (AchE) were maintained in hypoxia and activated during reoxygenation in both gibel carp and silver carp. AchE activity in fish brain correlates with locomotor activity (Rao et al., 2005; Venkateswara Rao et al., 2006), and activation of this enzyme might stimulate adaptive behaviors such as foraging or habitat search during post-hypoxic recovery of the cyprinid fish. Recent findings have demonstrated that raising acetylcholine both *in vivo* and *in vitro* lowers apoptosis and ROS brought on by hypoxia or ischemia in mammals (Miao et al., 2013). It is thus tempting to speculate that elevated AChE activity might play similar protective roles during reoxygenation in hypoxia-tolerant fish like gibel carp. This hypothesis requires further investigation.

### Metabolic shifts during hypoxia-reoxygenation in cyprinids

The adaptive physiological responses of hypoxia-tolerant organisms to oxygen fluctuations rely heavily on mitochondrial adjustments (Bickler and Leslie, 2007; Pamenter, 2014; Sokolova et al., 2019). While hypoxia-sensitive species experience mitochondrial respiration collapse during reoxygenation, hypoxia-tolerant species exhibit robust or enhanced mitochondrial respiration following an H/R cycle, which is essential for restoring energy homeostasis during recovery (Adzigbli et al., 2022a,b; Buck and Pamenter, 2006; Ivanina et al., 2016; Pamenter, 2014; Sokolov et al., 2021). However, this increased respiration can lead to the production of ROS and potential oxidative stress during hypoxia and reoxygenation. Our study showed that in gibel carp, CCO activity was upregulated in both the gill and liver during hypoxia, and further elevated in the gills during recovery. Similarly, another hypoxia-tolerant cyprinid, the goldfish *Carassius auratus*, exhibited increased CCO activity in its muscle after short-term intermittent hypoxia exposure, which was not sustained after long-term (30 days) hypoxia (Thoral et al., 2022). However, our study found that the CCO activity in the liver of gibel carp was suppressed during reoxygenation, which is in line with our previous conclusion that reoxygenation is more stressful for this species than hypoxia. Conversely, the more hypoxia-sensitive silver carp showed no evidence of change in CCO activity during H/R exposures in the gill, but instead exhibited a gradual decline in CCO activity during hypoxia and subsequent reoxygenation in the liver. CCO is an important regulatory enzyme in the electron transport chain that can serve as an index of the organism's and tissue aerobic capacity (Norin and Malte, 2012; Thoral et al., 2022). Elevated CCO activity is commonly linked to improved respiration and metabolic performance in fish (Guderley, 2004; Norin and Malte, 2012; Thoral et al., 2022). Hence, the hypoxia-induced upregulation of CCO activity in the gill and liver of gibel carp may contribute to its enhanced aerobic capacity and hypoxia tolerance. Moreover, the further increase in CCO activity in the gill of gibel carp during reoxygenation may support metabolic flux and aid in oxygen uptake during the recovery phase.

Interestingly, our study showed that SDH activity was suppressed during hypoxia in gibel carp liver (no data are available for the gill). Succinate accumulates during hypoxia in cyprinid fish (Dahl et al., 2021), and its oxidation generates high ROS production rates (Okoye et al., 2021; Quinlan et al., 2013). Therefore, decreasing the SDH:CCO activity ratio during hypoxia may enable gibel carp to sustain high oxidative flux while keeping ROS production at bay. However, during reoxygenation, this trend is reversed, leading to an elevated SDH:CCO activity ratio, heightened ROS generation, and an accumulation of lipid peroxidation products in the fish's liver. The sensitivity to oxidative stress during reoxygenation in the hypoxia-tolerant cyprinid species may be attributed to the dysregulation of the relative activities of different ETS complexes. Notably, the SDH:CCO activity ratio was consistently lower in all experimental treatments (0.02-0.08) in silver carp liver than in gibel carp (0.04-0.16), despite the former exhibiting higher levels of ROS production than the latter. This highlights the importance of considering the species-specific ETS composition when interpreting the implications of ETS stoichiometry for ROS production.

### Conclusions, limitations and outlook

Our study investigated the impact of short-term hypoxia on oxidative stress and metabolic biomarkers in two species of cyprinids, silver carp and gibel carp. The results showed that the hypoxic exposure had a significant effect on the oxidative stress and metabolic biomarkers in silver carp but had a lesser impact on gibel carp. However, both species experienced perturbations of metabolic balance during the subsequent reoxygenation indicating a high cost of post-hypoxic recovery in both species. It is notable that this sensitivity to reoxygenation was observed after a relatively short period of hypoxic exposure (1 h). Our study also revealed that species-specific differences were more strongly associated with oxidative stress status, whereas metabolic indices and nitrosative stress parameters were more relevant to the response to hypoxia-reoxygenation. These findings support the idea that antioxidant protection may have a limited role in gibel carp and that other mechanisms, such as metabolic adjustments, molecular chaperones, or protein quality-control mechanisms, may be more important (Falfushynska et al., 2016; Sokolov et al., 2021, 2019; Zhang et al., 2012). However, it is important to note that our study only examined two closely related species of cyprinids, limiting the scope for evolutionary or adaptive conclusions. Further comparative research across a broader range of species is required to determine the broader applicability of these findings.

## MATERIALS AND METHODS

### Animal care and experimental exposures

Males of gibel carp *Carassius gibelio* (Bloch, 1782) (average mass 95.1±6.1 g, average body length 16.2±3.1 cm) and silver carp *Hypophthalmichthys molitrix* (Valenciennes, 1844) (average mass 118.3±9.3 g, average body length 19.7±3.5 cm) were provided by a local aquaculture supplier (Ternopil Region, Ukraine). The fish were kept in 200-L tanks containing dechlorinated tap water at the Ternopil V. Hnatiuk National Pedagogical University (Ukraine). The fish were kept in a 12 h:12 h light:dark cycle and fed daily with commercial fish food (Aquarius, Kharkiv, Ukraine). The temperature of the water tanks was maintained at 19±1°C, the DO concentration was 7.5±0.2 mg L^−1^, and the pH was 7.55±0.05. Electrical conductivity, hardness, ammonia, nitrite, chloride concentrations and oxidizability of the water were tested daily using standard techniques, and the values did not exceed permissible levels for tap water or water in natural ponds for the maintenance of cultured fish (Bhatnagar and Devi, 2009). Fish were acclimated to laboratory conditions for 14 days prior to hypoxia treatment and fasted for 24 h before the experimental exposures. All experiments followed the guidelines for the laboratory animal welfare and were approved by the animal ethics committee of Ternopil V. Hnatiuk National Pedagogical University (protocol number 1; March 2020). To test the effects of hypoxia, the fish were placed into 20-L tanks (five fish per tank) at 19±0.3°C. Hypoxia was achieved by bubbling nitrogen through a submersible pump until the DO level of 2.0 mg L^−1^ (4.55 kPa) was achieved. DO less than 2 mg/L is considered the lethal oxygen threshold for many freshwater animals (Fondriest, 2023). The fish were kept for an hour in hypoxia, and the DO was maintained at 2.0 mg L^−1^ and continuously monitored using an oxymeter AZ-86021 (Taiwan). No fish lost its ability to maintain balance during the hypoxia exposure. Half of experimental fish were randomly selected and euthanized after 1 h of hypoxia (see ‘Tissue sampling’, below). The remaining fish were placed in the normoxic aquaria for 1 h to recover after hypoxia and sampled after 1 h of post-hypoxic reoxygenation. Control fish were maintained under the normoxic conditions (DO=7.5±0.2 mg L^−1^) throughout the experiment. Three replicate tanks with 5 fish each were used for each experimental treatment.

### Tissue sampling and extraction

The fish was anesthetized by clove oil and euthanized by rapid cervical transection followed by pithing. The blood was collected and centrifuged at 1500 ***g*** for 15 min to obtain the blood serum. The gills, brain and liver were collected and immediately shock frozen for further analyses. Tissue samples (1:10 w:v) were homogenized in ice-cold 0.1 M phosphate buffer (pH 7.4) containing 100 mM KCl, 1 mM ethylenediaminetetraacetic acid (EDTA) and 0.1 mM phenylmethylsulfonyl fluoride (PMSF) and centrifuged at 6000 ***g*** for 10 min at 4°C. Protein content of the serum and tissue homogenates was measured using the Lowry's method (1951) with bovine serum albumin as a standard.

All biochemical and enzymatic markers were measured using ultraviolet (UV)/Vis spectrophotometer U-Lab 101UV (China), Biorad Vis-plate reader (USA) and Multiskan™ FC Microplate Photometer (ThermoFisher Scientific, USA) (for absorbance-based assays) or the *f*-max microplate reader (Molecular Devices, USA) (for the fluorescence-based assays). Chemicals were purchased from Merck (Synbias, Ukraine) and Sigma Aldrich (USA) and were of the analytical grade or higher. Sample size (N) was 5 per group for all studied parameters.

### Antioxidants

The 2,2′-azinobis (3-ethylbenzothiazoline 6-sulfonate) (ABTS) assay (based on the reduction rate of the ABTS^+^ radical by cellular antioxidants) was used to measure total antioxidant capacity (TAC) in soluble protein fractions of gills and liver tissue homogenates (Katalinic et al., 2005). Trolox solutions were used as calibration standards. The decrease in ABTS+ absorbance at 734 nm was used to determine the ability of a biological sample to scavenge radicals and expressed in Trolox equivalents per g tissue.

Catalase (CAT) activity was assessed in the soluble protein fraction of the gills and liver tissue homogenates (1:10 w:v) by monitoring the decline in hydrogen peroxide at 240 nm in 0.1 M potassium phosphate buffer, pH 7.4 as described in Aebi et al. (1974). The extinction coefficient *ε*=40 M^−1^ cm^−1^ was used to calculate the specific CAT activity per mg soluble protein.

Total glutathione (GSH) level was measured in the deproteinized extracts of gills and liver tissue samples using the Ellman's reagent as a chromogen at 412 nm (Jollow et al., 1974). The GSH concentration was calculated using the molar extinction coefficient of 14.15×10^3^ M^−1^ cm^−1^ and expressed as µmol per g wet tissue mass.

The activity of glutathione-S-transferase (GST, EC 2.5.1.18) was determined in the soluble protein fraction of the gills and liver tissue homogenates using 1-chloro-2,4-dinitrobenzene (CDNB) as a substrate (Habig et al., 1974). The GST activity was assessed at 340 nm and expressed as nmol min^−1^ mg^−1^ protein.

### Oxidative and nitrosative stress markers

Lipid peroxidation (LPO) was determined in the gills and liver tissues homogenates by measuring the levels of thiobarbituric acid-reactive substances at 532 nm (Ohkawa et al., 1979). The LPO levels were calculated using the molar extinction coefficient of 1.56×10^5^ M^−1^ cm^−1^ and expressed as nmol g^−1^ wet tissue mass.

Protein carbonyls (PC) were determined in the gills and liver homogenates using the reaction of damaged proteins with 2,4-dinitrophenylhydrazine (DNPH) detected at 370 nm (Reznick and Packer, 1994). The PC concentrations were calculated using the molar extinction coefficient of 2.1×10^4^ M^−1^ cm^−1^ and expressed as µmol g^−1^ wet tissue mass.

The cytosolic fraction was extracted from the liver and gill tissue homogenates in 20 mM HEPES-sucrose lysis buffer (1:10, w:v). The reaction of the oxidative conversion of dihydrorhodamine 123 into fluorescent rhodamine 123 was carried out in the HEPES-containing media. Reactive oxygen species (ROS) was observed at 485 nm/535 nm (excitation/emission) (Viarengo et al., 1999) and expressed as the relative fluorescence units (RFU) g^−1^ wet tissue mass.

The tissue levels of the NO breakdown products (nitrite and nitrate) were measured spectrophotometrically at 540 nm following Griess reaction as a marker of reactive nitrogen species levels (Sessa et al., 1994). Vanadium (III) chloride was used as the reductant. A standard curve was prepared with sodium nitrite. The NO concentration was presented as nmol mg^−1^ protein.

The DNA strand breaks in the supernatant of the gills and liver tissue following the alkaline DNA precipitation method (Olive, 1988). The assay was carried out in the presence of 0.4 M NaCl, 4 mM sodium cholate and 0.1 M Tris (pH 9) to reduce the interference from sodium dodecyl sulphate traces (Bester et al., 1994). The concentration of the fluorescent product reflecting the DNA double strand breaks was determined at 360 nm/450 nm (excitation/emission) and expressed as the percentage of the alkaline-precipitated DNA to the total DNA in the sample.

### Mitochondrial markers

The activity of succinate dehydrogenase (SDH, E. C. 1.3.99.1) was determined by the ferricyanide method based on the oxidation of succinate to fumarate by potassium ferricyanide (King, 1967). The reaction was terminated by the addition of 20% trichloroacetic acid. The absorbance of the reaction product was measured at 420 nm, and the SDH activity expressed as µmol min^−1^ mg^−1^ protein.

Activity of cytochrome c oxidase (CCO, EC 7.1.1.9) was measured by monitoring the oxidation of reduced cytochrome c in the presence of lauryl maltoside (Racay et al., 2009). The reaction was started by addition of 1 mM reduced cytochrome c. The resulting chromophore was measured at 550 nm. The CCO activity was calculated using the molar extinction of 19.6 M^−1^ cm^−1^ at 550 nm for oxidized cytochrome c and expressed as nmol min^−1^ g^−1^ wet tissue.

Mitochondrial swelling, as a marker of mitochondria permeability transition pore opening, was measured in the suspension of freshly isolated mitochondria from the fish liver. The mitochondria were isolated from the crude homogenate of the liver tissue in 230 mmol L^−1^; mannitol 75 mmol L^−1^ sucrose; 20 mmol L^−1^ HEPES; 1 mmol L^−1^ EGTA, pH 7.4 which was centrifuged at 800 ***g*** for 10 min at 4°C to remove cell debris (Gerber et al., 2020). The resulting supernatant was centrifuged at 8000 ***g*** for 10 min at 4°C to pellet the mitochondria. The mitochondrial suspension was placed in the assay buffer containing 200 mM sucrose, 10 mM Tris- 3-(*N*-morpholino)propanesulfonic acid (MOPS), 5 mM α-ketoglutarate, 2 mM malate, 1 mM K_2_HPO_4_, 10 μM ethylene glycol-bis(β-aminoethyl ether)-*N*,*N*,*N*′,*N*′-tetraacetic acid - tris(hydroxymethyl)aminomethane (EGTA-Tris), pH 7.4. Swelling of mitochondria was determined in the absence and presence of CaCl_2_ by monitoring the decrease in light scattering at 540 nm (Jang and Javadov, 2017) and expressed as difference in decreasing of absorbance over time with and without CaCl_2_.

### Apoptotic marker

The activity of the apoptosis executor caspase 3 was measured at 405 nm using a chromogenic caspase-3 substrate acetyl-Asp-Glu-Val-Asp p-nitroanilide (Bonomini et al., 2004; Falfushynska et al., 2017). The caspase-3 activity was calculated using the molar extinction coefficient of 10.5 mM^−1^ cm^−1^ and expressed as nmol min^−1^ mg^−1^ protein.

### Autophagic markers

The activity of the lysosomal protease cathepsin D (EC 3.4.23.5) was assessed in the gills and liver tissue homogenates prepared in 0.25 M sucrose (1:2 w:v). The activity of cathepsin D was determined by measuring hemoglobin digestion at pH 3.2 (Barrett, 1967). The total cathepsin D activity was determined following Triton X-100 treatment, whereas free cathepsin D activity was evaluated in tissue homogenate without the addition of a detergent. The difference between the total and free activities was used to determine the lysosomal cathepsin D activity. The absorbance of the digestion product was determined at 280 nm using tyrosine as a standard. The cathepsin D activity was expressed as nmol tyrosine min^−1^ mg^−1^ soluble protein.

The Neutral Red Retention assay was used to evaluate the stability of the lysosomal membranes. The lysosome-enriched fraction was isolated from the liver tissue homogenates using differential centrifugation (Nazar et al., 2008). The lysosomal fraction was incubated for 2 h in Ringer's solution containing 0.004% (w:v) Neutral Red. The lysosomal-reach fractions were washed three times after exposure to remove any non-accumulated Neutral Red. In turn, the accumulated dye was extracted with ethanol/acetic acid (1:1 v:v) solution. The absorbance was measured at 550 nm, and the Neutral Red Retention was expressed as D_550_ mg^−1^ soluble protein (Falfushynska et al., 2019).

### Tissue damage markers

Cytotoxicity and tissue damage were assessed by the release of lactate dehydrogenase (LDH) into the blood serum (Casillas and Ames, 1986). A spectrophotometric protocol based on the conversion of pyruvate into lactate in the presence of NADH monitored at 340 nm was used to determine the LDH activity (EC 1.1.1.27) in blood serum samples (Bergmeyer and Bernt, 1974). The LDH activity was calculated using the molar extinction coefficient of 6.22×10^3^ M^−1^ cm ^−1^ for NADH and expressed as µmol min^−1^ ml^−1^ blood serum.

Acetylcholinesterase (AChE, EC 3.1.1.7) activity was measured in the soluble protein fraction of the brain tissue homogenates using acetylthiocholine as the substrate and the Ellman's reagent as a chromogen monitored at 412 nm (Ellman et al., 1961). The AChE activity calculated using the extinction coefficient of 14.15 mM^−1^ cm^−1^ and expressed as nmol min^−1^ g^−1^ tissue.

### Statistical data processing

The data were tested for normal distribution and homogeneity of variances using the Kolmogorov–Smirnov and Levine tests, respectively. For non-normally distributed variables, Box–Cox transformation was applied. Two-way ANOVA was used to test for the effects of predictors (biological species, hypoxia/reoxygenation stress) and their interactions on the studied response variables (Table S1). Tukey's honest significant difference (HSD) test was used as a post-hoc tests to determine whether there were any statistically significant differences between the means. To reduce the dimensionality of the multi-biomarker dataset and visualize the species-specific and H/R-induced differences in the integrated biomarker profiles, we used partial-least square discriminant analysis (PLS-DA) and heat map implemented in MetaboAnalyst 5.0 (Pang et al., 2021). Prior to PLS-DA and heat map analysis, biochemical and physiological indices were auto-scaled (mean-centered and divided by the standard deviation of each variable) to reduce the differences of scale between the studied variables. Data analysis was conducted using Statistica 12.0, MetaboAnalyst 5.0, and Excel for Windows-2019. Statistical significance was determined using a Type I error (P) threshold of 0.05. For all biochemical and molecular analyses, five biological replicates were used. Results are presented as means with standard errors of the mean (s.e.m.).

## Supplementary Material

10.1242/biolopen.060069_sup1 Supplementary information
