# Supplementary material for: Intermittent hypoxia differentially affects metabolic and oxidative stress responses in two species of cyprinid fish
Source: Biol Open. 2023 Sep 18;12(9):bio060069. doi: 10.1242/bio.060069 (PMC10537972; doi:10.1242/bio.060069)
Supplement: Supplementary information [file biolopen-12-060069-s1.pdf]

Table S1. Two-way ANOVA of the studied biological traits of gibel carp and silver carp exposed to normoxia, hypoxia, and reoxygenation.

	Fish species	Hypoxia/reoxygenation	Species×H/R
		**Liver**	
Total antioxidant capacity	**$F_{1,24}$=29.5, p<0.001**	$F_{2,24}$=0.84, p=0.44	$F_{2,24}$=2.48, p=0.104
Reactive oxygen species	**$F_{1,24}$=66.3, p<0.001**	$F_{2,24}$=0.51, p=0.61	**$F_{2,24}$=3.46, p=0.045**
DNA strand breaks	**$F_{1,24}$=14.3, p<0.001**	$F_{2,24}$=0.72, p=0.50	$F_{2,24}$=1.48, p=0.247
Glutathione total	$F_{1,24}$=2.17, p=0.154	$F_{2,24}$=1.10, p=0.35	$F_{2,24}$=1.40, p=0.265
Reactive nitrogen species	**$F_{1,24}$=66.6, p<0.001**	$F_{2,24}$=2.08, p=0.15	**$F_{2,24}$=7.30, p=0.004**
Glutathione-S-transferase	**$F_{1,24}$=68.5, p<0.001**	**$F_{2,24}$=4.51, p=0.02**	**$F_{2,24}$=8.35, p=0.002**
Catalase	**$F_{1,24}$=94.0, p<0.001**	$F_{2,24}$=2.37, p=0.12	**$F_{2,24}$=6.66, p=0.005**
Caspase 3	**$F_{1,24}$=32.5, p<0.001**	**$F_{2,24}$=4.57, p=0.02**	**$F_{2,24}$=11.57, p<0.001**
TBARS	**$F_{1,24}$=15.3 p<0.001**	**$F_{2,24}$=25.2, p<0.001**	**$F_{2,24}$=76.08, p<0.001**
Protein carbonyls	**$F_{1,24}$=46.8, p<0.001**	$F_{2,24}$=0.67, p=0.52	$F_{2,24}$=2.48, p=0.104
Cathepsin D total	**$F_{1,24}$=150.5, p<0.001**	**$F_{2,24}$=12.3, p<0.001**	**$F_{2,24}$=9.23, p=0.001**
Cathepsin D free	**$F_{1,24}$=89.7, p<0.001**	$F_{2,24}$=2.96, p=0.07	**$F_{2,24}$=6.68, p=0.005**
Cathepsin D, lysosomal/free	**$F_{1,24}$=5.8, p=0.022**	**$F_{2,24}$=11.3, p<0.001**	**$F_{2,24}$=11.1, p<0.001**
Cytochrome c oxidase	**$F_{1,24}$=30.2, p<0.001**	**$F_{2,24}$=32.3, p<0.001**	**$F_{2,24}$=4.66, p=0.020**
Succinate dehydrogenase	$F_{1,24}$=1.0, p=0.758	$F_{2,24}$=17.4, p<0.001	$F_{2,24}$=0.05, p=0.947
Mitochondria swelling	$F_{1,24}$=3.6, p=0.068	**$F_{2,24}$=20.0, p<0.001**	$F_{2,24}$=0.09, p=0.915
Neutral red retention	**$F_{1,24}$=22.6, p<0.001**	**$F_{2,24}$=9.52, p<0.001**	**$F_{2,24}$=6.15, p=0.007**

Biology Open • Supplementary information

**Gills**			
Total antioxidant capacity	**$F_{1,24}$=191.6, p<0.001**	**$F_{2,24}$=28.8, p<0.001**	**$F_{2,24}$=38.3, p<0.001**
Reactive oxygen species	**$F_{1,24}$=226.5, p<0.001**	**$F_{2,24}$=22.2, p<0.001**	$F_{2,24}$=1.01, p=0.379
DNA strand breaks	**$F_{1,24}$=143.2, p<0.001**	$F_{2,24}$=0.94, p=0.41	$F_{2,24}$=3.31, p=0.051
Glutathione total	**$F_{1,24}$=5.8, p=0.023**	**$F_{2,24}$=5.21, p=0.01**	$F_{2,24}$=2.25, p=0.127
Reactive nitrogen species	**$F_{1,24}$=7.2, p=0.012**	**$F_{2,24}$=10.98, p<0.001**	**$F_{2,24}$=7.8, p=0.002**
Glutathione-S-transferase	**$F_{1,24}$=13.1, p<0.001**	**$F_{2,24}$=14.56, p<0.001**	**$F_{2,24}$=13.5, p<0.001**
Catalase	**$F_{1,24}$=37.8, p<0.001**	$F_{2,24}$=0.55, p=0.582	**$F_{2,24}$=11.6, p<0.001**
Caspase 3	**$F_{1,24}$=13.5, p=0.001**	$F_{2,24}$=0.40, p=0.672	**$F_{2,24}$=11.0, p<0.001**
TBARS	**$F_{1,24}$=37.4, p<0.001**	**$F_{2,24}$=12.7, p<0.001**	**$F_{2,24}$=40.2, p<0.001**
Protein carbonyls	**$F_{1,24}$=6.1, p=0.021**	$F_{2,24}$=2.41, p=0.111	$F_{2,24}$=0.04, p=0.962
Cytochrome c oxidase	$F_{1,24}$=0.27, p=0.609	**$F_{2,24}$=7.75, p=0.003**	**$F_{2,24}$=10.5, p<0.001**
**Brain**			
AChE	$F_{1,24}$=2.88, p=0.102	**$F_{2,24}$=18.8, p<0.001**	$F_{2,24}$=0.94, p=0.406
**Blood**			
LDH	**$F_{1,24}$=9.32, p=0.005**	**$F_{2,24}$=38.8, p<0.001**	**$F_{2,24}$=8.3, p=0.002**

Biology Open • Supplementary information

Table S2. Loadings of the studied biological traits of gibel carp and silver carp exposed to normoxia, hypoxia, and reoxygenation on the three first components in the partial least square discriminant analysis.

	Component 1 (34.9% variation)	Component 2 (17.4% variation)	Component 3 (7.2% variation)
Liver			
Total antioxidant capacity	0.2333	-0.02472	0.11326
Reactive oxygen species	0.25129	0.009493	-0.09041
DNA strand breaks	0.19279	-0.02881	0.10015
Glutathione total	0.078804	0.16134	-0.14948
Reactive nitrogen species	-0.26776	0.13602	-0.10722
Glutathione-S-transferase	-0.25713	0.13872	-0.17484
Catalase	-0.28195	0.12672	-0.11664
Caspase 3	-0.18048	0.041957	0.46294
TBARS	0.082002	0.16099	-0.30776
Protein carbonyls	0.2485	-0.07261	-0.08734
Cathepsin D total	-0.26921	0.18181	-0.19895
Cathepsin D free	-0.26771	0.15926	-0.11674
Cathepsin D, lysosomal/free	0.12248	0.18364	-0.1006
Cytochrome c oxidase	0.12664	-0.34162	0.052286
Succinate dehydrogenase	0.013544	0.27987	-0.22894
Mitochondria swelling	-0.052	0.35317	0.052945
Neutral red retention	-0.19753	0.092115	-0.49966
Gills			
Total antioxidant capacity	0.24903	0.15231	0.029424
Reactive oxygen species	-0.24547	0.18766	0.19275
DNA strand breaks	-0.26239	0.051572	0.15776
Glutathione total	0.139	0.014893	0.26557
Reactive nitrogen species	0.072259	-0.21584	-0.44802
Glutathione-S-transferase	-0.14435	-0.24859	-0.17396

Catalase	-0.21522	0.084297	-0.05102
Caspase 3	0.13089	-0.06042	-0.18221
TBARS	0.15139	0.20103	-0.24357
Protein carbonyls	0.14101	0.048851	-0.30462
Cytochrome c oxidase	0.020225	0.17308	0.10177
Brain			
AChE	0.1079	0.30128	-0.27278
Blood			
LDH	-0.05741	0.38649	0.13323

Biology Open • Supplementary information
